# The front-page filter in bibliometric research: implications for retrieval precision and methodological attribution

**DOI:** 10.3389/fnagi.2026.1903685

**Published:** 2026-07-10

**Authors:** Yuh-Shan Ho, Nikolaos Christidis

**Affiliations:** 1 CT HO Trend, Taipei, Taiwan; 2 Division of Oral Rehabilitation, Department of Dental Medicine, Karolinska Institutet, Huddinge, Sweden

**Keywords:** bibliometrics, front-page filter, methodological attribution, retrieval precision, Web of Science

## 1 Introduction

As bibliometric research continues to expand across the health sciences, the precision of data retrieval remains a critical determinant of study validity (Donthu et al., 2021; Moral-Muñoz et al., 2020). One methodological development that has received increasing attention is the “front page” filter, which restricts retrieval to information contained in the Title (TI), Abstract (AB), and Author Keywords (AK) fields. This approach has been proposed as a more precise alternative to conventional Topic (TS) searches that include *Keywords Plus* (KP). Beyond issues of retrieval accuracy, discussion of the front-page filter raises broader questions regarding retrieval strategy selection, methodological transparency, and scholarly attribution. While bibliometric studies frequently discuss database selection, indicators, and analytical tools, less attention has been given to how retrieval-field selection itself may influence dataset composition, reproducibility, and interpretation of results (Donthu et al., 2021; Moral-Muñoz et al., 2020). This opinion article aims to discuss the role of the front-page filter within this broader methodological context and to highlight the importance of transparent reporting and attribution of methodological developments.

## 2 The front-page filter and its methodological implications

### 2.1 Methodological development and retrieval precision

Retrieval precision has long been recognized as an important methodological consideration in bibliometric research (Donthu et al., 2021). The front-page filter represents one such methodological development and has been empirically evaluated in several previous investigations (Wang and Ho, 2011; Fu et al., 2012). The current TI-AB-AK framework is not merely a search refinement but a documented methodological contribution that has been described and further developed in the bibliometric literature (Usman and Ho, 2021). Consistent citation of these foundational studies

would strengthen methodological transparency and provide appropriate recognition of the framework's development.

### 2.2 Limitations of topic-based retrieval

Historically, many Web of Science retrieval strategies relied on the Topic (TS) field because of database interface limitations and common search practices. The TS field includes *Keywords Plus* (KP), a retrieval component generated from cited references rather than from author-supplied descriptors (Garfield, 1990). A recognized limitation of TS-based retrieval is the inclusion of records in which the search term is peripheral to the main focus of the study, thereby reducing dataset precision. Such imprecision may affect the validity, reproducibility, and interpretability of bibliometric findings.

### 2.3 Development and standardization of the front-page filter

The front-page filter was developed to address these limitations by restricting retrieval to author-defined metadata, namely the Title (TI), Abstract (AB), and Author Keywords (AK) fields. By excluding *Keywords Plus*, this framework places greater emphasis on author-defined relevance and reduces the inclusion of algorithm-generated retrieval noise. Initially proposed by (Wang and Ho 2011) and subsequently evaluated in SCI-EXPANDED studies (Fu et al., 2012), the approach evolved into the current TI-AB-AK framework described by (Usman and Ho 2021). Its application in medical and health-science bibliometric studies (Yang et al., 2022; Ho et al., 2023; Peng et al., 2024; Rao et al., 2025; Christidis and Ho, 2026) suggests that it can serve as a useful approach for improving retrieval precision in certain research contexts. However, its suitability may depend on the objectives of the study and the balance sought between retrieval precision and retrieval comprehensiveness.

### 2.4 Empirical implications for bibliometric research

Comparative studies have demonstrated that omission of the front-page filter may result in substantial differences in bibliometric outputs, particularly within the health sciences. Reliance on TS-based retrieval may introduce peripheral literature that obscures central research themes and influences the interpretation of publication trends. The substantial differences in publication counts presented in Table 1 illustrate the potential impact of retrieval strategy on bibliometric outcomes and support the value of high-precision search approaches. At the same time, higher precision may be accompanied by lower recall. Restricting retrieval to title, abstract, and author keywords may exclude publications in which the topic is relevant but not explicitly represented in these fields. Consequently, the optimal retrieval strategy may vary depending on the purpose of the bibliometric analysis and the desired balance between precision and comprehensiveness. Similar trade-offs between precision and comprehensiveness have been discussed more broadly in methodological studies of bibliometric retrieval and dataset construction (Donthu et al., 2021).

**Table 1:** Comparative analysis of retrieval deviation: topic (TS) search vs. the “front page” filter (TI-AB-AK) across medical research domains.

Research topic	TS search (records)	Front page (records)	% Difference (“noise”)
The top 100 most-cited articles on kyphoplasty and vertebroplasty (Ho, 2020a)	4,695	2,778	59%
Radiotherapy for gliomas (Ho, 2023)	5,225	2,876	55%
Stem cell therapy for spinal cord injury (Ho, 2020b)	3,863	1,991	52%
Macrophage polarization (Ho, 2019)	3,064	1,507	49%
Immunological factors in cirrhosis diseases (Ho and Ouchi, 2025)	3,050	1,446	47%
Intestinal barrier in inflammatory bowel disease (Ho and Ouchi, 2025)	4,585	1,422	31%
The relationship between gastric cancer and depression (Ho and Ouchi, 2025)	282	82	29%
Intestinal microcirculation (Ho and Ouchi, 2025)	1,343	391	29%
Treatment for nonalcoholic fatty liver disease (Ho and Ouchi, 2025)	13,133	1,853	14%
Artificial intelligence and endoscopy in digestive diseases (Ho and Ouchi, 2025)	4,197	377	9.0%

### 2.5 Attribution of methodological contributions

Applications of the TI-AB-AK framework have appeared in an increasing number of bibliometric studies (Wu et al., 2025; Yang et al., 2026). However, citation of the methodological studies that established and refined this framework has not always been uniform across the literature. As (Noè and Batten 2006) emphasized, appropriate acknowledgment of conceptual and methodological contributions remains an important aspect of research integrity. By contrast, other authors have appropriately cited the methodological foundations of the TI-AB-AK framework when applying similar approaches (Peng et al., 2024; Hu et al., 2024).

The evidence discussed here suggests that the front-page filter represents one approach for improving dataset precision in bibliometric research. Nevertheless, inconsistent citation of foundational methodological studies may weaken intellectual continuity and obscure the developmental trajectory of widely adopted bibliometric approaches. Maintaining clear links between methodological innovations and their original empirical descriptions promotes transparency, reproducibility, and appropriate scholarly recognition. As bibliometric methods continue to evolve, consistent reporting and citation of methodological developments will remain essential. Greater attention to methodological attribution may strengthen both reproducibility and scholarly continuity within the bibliometric literature.

## 3 Discussion

The continued growth of bibliometric research has increased the importance of transparent and reproducible retrieval strategies. The front-page filter represents one methodological option intended to improve retrieval precision by emphasizing author-defined content rather than algorithmically generated indexing terms. The retrieval differences summarized in Table 1 illustrate that search-field selection may substantially influence bibliometric datasets and subsequent interpretations. However, retrieval precision should not be viewed as the sole criterion for evaluating search strategies. A more restrictive search may reduce the inclusion of peripheral records but may also exclude potentially relevant publications, reflecting the well-recognized trade-off between precision and recall in information retrieval and literature searching (Falagas et al., 2008; Bramer et al., 2017). Consequently, no single retrieval approach can be considered universally optimal. Rather, the appropriateness of a retrieval strategy should be evaluated in relation to the objectives of the study, the characteristics of the research field, and the desired balance between precision and comprehensiveness. The broader implication is that bibliometric findings may be influenced not only by analytical techniques but also by the retrieval fields selected during dataset construction. his observation aligns with broader methodological discussions in bibliometric research, where data collection, preprocessing, and analytical choices are all recognized as factors that may influence study outcomes (Aria and Cuccurullo, 2017; Donthu et al., 2021; Moral-Muñoz et al., 2020). Greater transparency in reporting retrieval decisions would improve reproducibility and facilitate comparisons across studies (Zupic and Čater, 2015; Donthu et al., 2021). Future bibliometric investigations could benefit from sensitivity analyses comparing alternative retrieval strategies, including TS-based and TI-AB-AK-based approaches, as methodological choices during dataset construction may substantially influence subsequent bibliometric outcomes (Hernandez-Torrano et al., 2020; Donthu et al., 2021). Such comparisons may help researchers better understand how retrieval choices affect publication counts, thematic structures, citation patterns, and overall conclusions. Finally, methodological attribution remains an important aspect of research integrity. Appropriate citation of methodological developments helps maintain intellectual continuity, enables readers to trace the evolution of bibliometric practices, and promotes transparency in methodological reporting.
